# Characterization of Major Histocompatibility Complex (MHC) DRB Exon 2 and DRA Exon 3 Fragments in a Primary Terrestrial Rabies Vector (*Procyon lotor*)

**DOI:** 10.1371/journal.pone.0012066

**Published:** 2010-08-10

**Authors:** Sarrah Castillo, Vythegi Srithayakumar, Vanessa Meunier, Christopher J. Kyle

**Affiliations:** 1 Environmental and Life Sciences Gradate Program, Trent University, Peterborough, Ontario, Canada; 2 Forensic Science Department, Trent University, Peterborough, Ontario, Canada; 3 Natural Resources DNA Profiling and Forensics Centre, Trent University, Peterborough, Ontario, Canada; Smithsonian Institution National Zoological Park, United States of America

## Abstract

The major histocompatibility complex (MHC) presents a unique system to explore links between genetic diversity and pathogens, as diversity within MHC is maintained in part by pathogen driven selection. While the majority of wildlife MHC studies have investigated species that are of conservation concern, here we characterize MHC variation in a common and broadly distributed species, the North American raccoon (*Procyon lotor*). Raccoons host an array of broadly distributed wildlife diseases (e.g., canine distemper, parvovirus and raccoon rabies virus) and present important human health risks as they persist in high densities and in close proximity to humans and livestock. To further explore how genetic variation influences the spread and maintenance of disease in raccoons we characterized a fragment of MHC class II DRA exon 3 (250bp) and DRB exon 2 (228 bp). MHC DRA was found to be functionally monomorphic in the 32 individuals screened; whereas DRB exon 2 revealed 66 unique alleles among the 246 individuals screened. Between two and four alleles were observed in each individual suggesting we were amplifying a duplicated DRB locus. Nucleotide differences between DRB alleles ranged from 1 to 36 bp (0.4–15.8% divergence) and translated into 1 to 21 (1.3–27.6% divergence) amino acid differences. We detected a significant excess of nonsynonymous substitutions at the peptide binding region (P = 0.005), indicating that DRB exon 2 in raccoons has been influenced by positive selection. These data will form the basis of continued analyses into the spatial and temporal relationship of the raccoon rabies virus and the immunogenetic response in its primary host.

## Introduction

Genetic variation has been associated with resistance to pathogens; however, studies have primarily screened neutral molecular markers to assess levels of genetic diversity [e.g., [1], –[3]] despite their inability to reveal patterns of adaptive selection [e.g., 4]. Studying functional genetic markers, such as those within the major histocompatibility complex (MHC), provides an opportunity to assess genetic variation directly associated with adaptive selection [2], [3]. MHC is a multi-gene family, consisting of two tightly linked subclasses in birds and mammals, which play a vital role in the initiation of the immune response [5]–[7]. MHC class I molecules are responsible for recognition of intracellular pathogens such as viruses and cancer cells [2], [8], whereas class II molecules are responsible for recognition of extracellular pathogens such as bacteria and nematodes [8]. Given its immunological capabilities, MHC provides a genetic system to study disease dynamics in vertebrates [9]. MHC is one of the most polymorphic complexes of the vertebrate genome, with the majority of the polymorphism confined to the functionally important peptide binding region (PBR) [5], which bind peptides and presents them to T-cells, thereby activating the necessary immune response [3]. The PBR most often displays higher rates of nonsynonymous substitutions (amino acid change) than synonymous substitutions (same amino acid) as it allows for binding of a greater number of peptides [9], [10]. A number of hypotheses have been proposed to explain how the high levels of diversity at MHC are maintained, including overdominant selection, pathogen driven selection, maternal-fetal interactions and mate choice [8], [11], [12,]. However, a combination of different selection methods may be responsible for the extent of polymorphism observed and maintained within MHC [9].

Generally, investigations of MHC have focused on wildlife species of conservation concern that have experienced recent population reductions due to factors such as emerging infectious disease, and negative anthropogenic influences (e.g., common frog [13] Eurasian beaver [14], lemur [10], chacma baboon [15], sea lion [16], giant panda [17] and European mink [18]). The objective of this study was to characterize the DR region of MHC class II in a common and widespread wildlife species, the North American raccoon (*Procyon lotor*). Raccoons present a particularly interesting system to study MHC variation in mammals as they are broadly distributed across much of North America and are a host species to a number of pathogens and parasites (e.g., canine distemper virus, canine adenovirus, feline parvovirus, and rabies) [19] that can be transmitted to other wildlife, agricultural animals, and domestic animals [19]. Large bodies of water and large mountain ranges (e.g., Mississippi River and Appalachian Mountains) have been proposed as physiographic barriers to the movement of raccoons [20], [21]; however, raccoons generally lack strong patterns of genetic structure over broad geographic ranges. Bi-parentally inherited neutral markers show limited genetic structure of raccoons across North America, with slight structure found on a small spatial scale across large rivers (e.g., Niagara River) [22]. Maternally inherited neutral markers (mtDNA) show slightly stronger genetic structure in raccoons where there are three main lineages in North America [23], which are closely related to a previously considered separate species, the West-Indies raccoon [24]. The general lack of distinct raccoon populations throughout North America have been attributed to extensive gene flow, high population densities and long distance dispersal, and may have influenced the speed at which diseases are transmitted in this wildlife vector [25]. This study will add an additional dimension to our understanding of raccoon genetics by characterizing the DR region of MHC class II, which allows us to investigate the link between disease and the immune response. We examined two loci within the DR region of MHC class II (DRA and DRB), with focus on the second exon of DRB where the functionally important PBR resides [8] which has been previously studied in numerous wildlife species. This study will provide a baseline from which to expand our exploration of MHC in conjunction with wildlife diseases, demographic processes, and other selective forces.

## Materials and Methods

### Sample collection, DNA extraction and quantification

Samples were obtained from raccoons along the eastern seaboard of North America and consisted of a subset of those previously used for subspecific designation [23]. We chose four distinct geographic regions to study (Ontario (ON), New York (NY), Alabama/Georgia (AL/GA) and Florida (FL)), which differed in their exposure time to rabies. Samples were provided from a number of agencies including; Canadian Food Inspection Agency (CFIA), Center for Disease Control (CDC), New York Department of Health (NYDOH), Ontario Ministry of Natural Resources (OMNR), and United States Department of Agriculture-Wildlife Services (USDA-WS). Samples consisted of rabies positive and rabies negative individuals. Samples obtained from the CFIA, CDC and NYDOH were brain tissue samples from surveillance and rabies positive samples whereas samples obtained from OMNR and USDA-WS consisted of hair pulls from live trapped animals acquired during control programs, or muscle tissue.

DNA extraction methods were as per Cullingham et al. [23]. Briefly, samples were dissolved in 1× lysis buffer and 600 U/ml proteinase K. DNA extractions were carried out using an automated 96-well plate magnetic bead procedure on an Evolution P3 (Perkin Elmer, Waltham, USA) (May 2005) and quantified using PicoGreen® (Invitrogen, Burlington, Canada).

### PCR amplification and cloning procedure

We initially screened a 635bp fragment of MHC II DRA (exon 3–4) in 32 individuals, using the forward primer DRA U1291 (CCCGTGGAACTGGGAGAGC) and reverse primer DRA L1512 (CYRCATTCTCTGTKGTCTCTG) [16]. Polymerase chain reaction (PCR) was performed on a PTC-0220/PTC-0221 Thermocycler (Bio-Rad, Hercules, CA) using 10ng of DNA, 1× PCR buffer (Invitrogen, Carlsbad, CA), 0.45mM MgCl_2_, 1.5 mM of each dNTP, 0.3uM of each primer, 0.6 mM of bovine serum albumin (Sigma, St. Louis, MO), and 0.2 U/µl *Taq* DNA polymerase (Invitrogen, Carlsbad, CA), and double distilled water in a 15 µl reaction. PCR conditions started with an initial denaturation of 95°C for 11 min, followed by 35 cycles of the following steps: 94°C for 1 min, 59°C for 1 min and 72°C for 1 min, with a final extension of 45 min at 60°C. Visualization of amplified product was performed on an agarose gel stained with ethidium bromide. Amplified products were ligated into a vector and transformed into cells using pCR®2.1-TOPO vector, and TOP10 chemically competent cells following the procedure outlined in the TOPO TA cloning kit (Invitrogen, Carlsbad, CA) with the following modifications: 0.8µl of vector, and an incubation time (PCR product inserted into vector) of 30 min at room temperature. Following overnight incubation, sterile toothpicks were used to pick clones that were added to 50 µl of 0.1× TE_0.1_. Clones were boiled (10 min at 100°C) and 5–15 clones were amplified to confirm insertion using the primer set M13F (GTAAAACGACGGCCAG) and M13R (CAGGAAACAGCTATGAC) (Invitrogen, Carlsbad, CA). Amplification consisted of 2 µl of cloned produced, 1× PCR buffer, 0.04 mM of each dNTP, 1.5 mM MgCl_2_, 0.2 uM of each primer, 0.05 U/µl of *Taq* DNA polymerase and double distilled water in a 10µL reaction. Cycling conditions consisted of an initial denaturation at 95°C for 5 min, followed by 30 cycles of 95°C for 30 sec, 55°C for 30 sec, 72°C for 30 sec, and a final extension of 2 min at 72°C. Successfully inserted clones were purified for sequencing using ExoSap-IT (New England Biolabs Inc, Ipswich, MA) following the manufacturer's instructions. Sequencing using the M13F primer was carried out using the BigDye® Terminator v3.1 Cycle Sequencing Kit and the resulting fragments were analyzed on an ABI 3730 DNA Analyzer (Applied Biosystems, Foster City, CA). Fragments were visually inspected, corrected and aligned manually to other species and to each other, using MEGA version 4 [26]. Nomenclature rules set by Klein et al. [27] were followed for designating DR allele names.

In addition, we amplified a 228 base pair fragment of MHC II DRB exon 2, in 246 individuals, using the forward primer DRB-5c (TCAATGGGACGGAGCGGGTGC) [28] and reverse primer DRB-3c (CCGCTGCACAGTGAAACTCTC) [29]. Polymerase chain reaction (PCR) was performed using 10ng of DNA, 1× PCR buffer (Invitrogen, Carlsbad, CA), Q-Solution (Quiagen, Mississauga, Ontario), 1.5mM MgCl_2_, 0.2 mM of each dNTP, 0.45uM of each primer, and 0.05 U/µl *Taq* DNA polymerase (Invitrogen, Carlsbad, CA), and double distilled water in a 12 µl reaction. PCR conditions included an initial denaturation for 5 min at 94°C, followed by 34 cycles of the following steps: 94°C for 30 sec, 56°C for 1 min and 72°C for 1 min, with a final extension of 45 min at 60°C. Visualization and sequencing procedures were as outlined above for DRA. The cloning procedure differed slightly in the number of clones that were picked (20–30 clones/sample) and number of clones amplified (25 clones/sample).

### RNA Isolation

The expression of MHC DRB exon 2 was explored through RNA screening for transcription of the gene from fresh blood of a single raccoon from Ontario. RNA was isolated using the total RNA purification kit (Norgen Biotek Corp, Thorold, ON) following the manufacturer's instructions. Extracted RNA was further treated with DNase enzyme (New England Biolabs Inc, Ipswich, MA) according to manufacturer's protocol to remove any residual DNA and was cleaned using an isopropanol precipitation. cDNA was constructed using ThermoScript RT-PCR system (Invitrogen, Carlsbad, CA) following manufacturer's instructions. cDNA was constructed using gene specific primers and the expression of the gene was assumed confirmed by presence of band at ∼200 bp on an agarose gel.

### Analysis

A number of molecular techniques have been used to characterize MHC in mammals including DGGE [16], SSCP [30] and RSCA [31] in addition to cloning [32]. Upon initial characterization of MHC DRB exon 2 in raccoons we determined that many of the alleles differed by as little as one nucleotide, which produced different amino acid sequences. In addition, the total number of alleles increased as we augmented the number of individuals and geographic regions screened. Based on the aforementioned, we determined that cloning was the most appropriate method to use for this study in order to directly capture all the variation at MHC. Given the potential for cloning errors, which may result in recombinant alleles, singleton mutations, as well as non-target DNA incorporation during cloning [33], conservative criteria were used to confirm the presence of alleles. Any sequences showing singletons from *Taq* error and recombinations were immediately discarded. Sequences from clones were confirmed as alleles when they were seen in more than one clone from multiple individuals [34]. Sequences observed in more than one clone, but only from a single individual were confirmed as alleles through a second independent amplification and cloning procedure. Upon visual analysis of sequences it was determined that we were amplifying a duplicated locus, with each individual having between two and four alleles. This led to an increased number of clones that needed to be sequenced. Using a homogeneous discrete time Markov Chain (Tij = P{X_n+1_ = j|X_n_ = i}) [35] we determined that 16 clones needed to be sequenced to visualize all four alleles with a confidence interval of 96%. In the 16 clones per individual we saw evidence of *Taq* errors and chimers. We excluded all sequences that presented these types of artefacts leading to approximately 13 clones/individual showing redundancy, leaving an 85% chance of visualizing all alleles/individual.

We attempted to separate the alleles into their respective loci by using individuals presumed homozygous at each locus; however, many of the alleles observed in high frequencies appeared to be shared between the loci making it difficult to assign alleles to a specific locus. Further analysis was performed to supertype alleles [reviewed in 4]. Supertyping simplifies data analysis and interpretation of results by reducing sampling error and strengthening statistical relationships. This approach has been used in humans [36], as well as other mammals (e.g., lemurs) [4] by classifying MHC alleles to supertypes based on similar antigen-binding sites, structural similarities, and polarities [4]. We supertyped alleles based on common amino acids at the peptide binding region, however these criteria did not result in fewer types and therefore analyses were performed on all alleles.

Average pairwise nucleotide distances (Kimura 2 parameter model or K2P), Poisson-corrected amino acid distances and average rate of synonymous (d_S_) and nonsynonymous (d_N_) substitutions per site were computed in MEGA 4 [26] using the modified Nei-Gojobori method with the Jukes-Cantor correction for multiple substitutions [37]. Standard errors of the preceding calculations were obtained through 1000 bootstrap replicates. To test methods of selection acting on exon 2 of DRB in raccoons, rates of d_N_ and d_S_ were calculated both under models of neutrality and positive selection using a one tailed Z-test performed in MEGA. The rates of d_N_ and d_S_ were calculated separately for all amino acid positions (all sites), only peptide binding regions (PBR) and only non-PBR. The putative PBR was determined in concordance with the human MHC II molecular structure [38], [39].

A phylogenetic tree was constructed with Mr.Bayes, using Bayesian inference [40], [41], with the best-fit model of nucleotide substitution (F81+I+G) indicated by the likelihood ratio test of JModel test [42], [43]. Analyses were run for 80×10^7^ generations, sampling every 5000 generations. Branch support of phylogenies was assessed through Markov Chain Monte Carlo methodology. Outgroups of sea lion, European mink, and giant panda were chosen based on identity to other species MHC DRB exon 2 and similar carnivore species. In addition, a neighbour-joining tree was constructed from K2P nucleotide distance in MEGA with 100000 bootstraps. All trees used the same outgroups (sea lion, European mink and giant panda).

## Results

This study characterized two fragments of the MHC class II DR region in a large number of raccoons from different regions of North America. Initially, characterization of MHC DRA was performed on 32 individuals. Both intron and exon were amplified using the DRA primers, but we were only able to align exon 3 (250bp) with other known DRA sequences of sea lions (*Zaca*-DRA*03) [16], which was chosen based on its high similarity (96%) [44]. One to three alleles were observed per individual indicating that our primers were likely amplifying two loci. We found a total of three alleles (Genbank Accession HM589039–HM589041) in the 32 individuals screened. The nucleotide substitutions between the alleles were synonymous and translated into a single amino acid sequence indicating that DRA exon 3 is functionally monomorphic in raccoons; therefore no further analyses were performed using this marker.

MHC DRB exon 2 was screened in 246 individuals from four geographic regions (Table 1). Between two and four alleles were identified in each individual indicating we were likely amplifying a duplicated locus. A total of 66 unique alleles were detected among the 246 individuals analyzed (GenBank Accession GU388312–GU388377; Table 2). Of the 66 unique alleles, 58 were observed in more than one individual, whereas the remaining 8 were only seen in one individual but were observed in two or more clones. Confirmation of these 8 alleles was further assessed through a separate independent PCR and cloning procedure. Of the 228 nucleotides, 54 (23.7%) were variable as were 27 of the 75 (36.0%) amino acid positions. The number of pairwise nucleotide differences between pairs of alleles ranged from 1(6 pairs of alleles) to 36 (allele *Prlo*-DRB*31 vs. allele *Prlo*-DRB*80) and the number of amino acids differences ranged from 1 (19 pairs of alleles) to 21 (*Prlo*-DRB*31 vs. *Prlo*-DRB*80). There were no insertion/deletions or premature stop codons detected in DRB exon 2 in raccoons, suggesting it is functional. Functionality was further assessed through screening for transcription of DRB exon 2 in the RNA using RT PCR.

**Table 1:** Number of individuals and geographic locations of MHC DRB exon 2 alleles.

Allele	Number of individuals	Geographic location
*Prlo*-DRB*01	19	ON, NY, AL/GA, FL
*Prlo*-DRB*02	11	ON, NY, AL/GA
*Prlo*-DRB*03	8	ON, NY
*Prlo*-DRB*04	59	ON, NY, AL/GA, FL
*Prlo*-DRB*05	10	ON, NY
*Prlo*-DRB*06	20	ON, NY, AL/GA, FL
*Prlo*-DRB*07	66	ON, NY, AL/GA, FL
*Prlo*-DRB*08	2	ON, NY
*Prlo*-DRB*09	3	ON
*Prlo*-DRB*10	11	ON, NY, AL/GA, FL
*Prlo*-DRB*11	26	ON, NY, AL/GA, FL
*Prlo*-DRB*12	7	ON, NY, FL
*Prlo*-DRB*13	5	ON, NY, FL
*Prlo*-DRB*14	13	ON, NY, AL/GA
*Prlo*-DRB*15	4	ON, NY, AL/GA, FL
*Prlo*-DRB*16	18	ON, NY, AL/GA, FL
*Prlo*-DRB*17	1	ON
*Prlo*-DRB*18	3	ON, AL/GA
*Prlo*-DRB*19	62	ON, NY, AL/GA, FL
*Prlo*-DRB*20	28	ON, NY, FL
*Prlo*-DRB*21	7	ON, NY
*Prlo*-DRB*22	3	ON, FL
*Prlo*-DRB*24	15	ON, NY, AL/GA, FL
*Prlo*-DRB*25	11	ON, NY, AL/GA
*Prlo*-DRB*26	11	ON, NY
*Prlo*-DRB*27	8	ON, NY, AL/GA
*Prlo*-DRB*28	8	NY, AL/GA, FL
*Prlo*-DRB*29	3	NY, FL
*Prlo*-DRB*30	12	NY, FL
*Prlo*-DRB*31	17	NY, AL/GA, FL
*Prlo*-DRB*32	1	NY
*Prlo*-DRB*34	15	NY, AL/GA, FL
*Prlo*-DRB*42	24	ON, NY, AL/GA, FL
*Prlo*-DRB*43	5	ON, FL
*Prlo*-DRB*47	56	ON, NY, AL/GA, FL
*Prlo*-DRB*48	1	FL
*Prlo*-DRB*49	13	ON, NY, AL/GA
*Prlo*-DRB*50	13	FL
*Prlo*-DRB*51	4	NY, FL
*Prlo*-DRB*52	4	ON, FL
*Prlo*-DRB*53	4	ON, NY
*Prlo*-DRB*54	7	FL
*Prlo*-DRB*55	6	NY, AL/GA, FL
*Prlo*-DRB*56	12	NY, AL/GA, FL
*Prlo*-DRB*57	45	AL/GA, FL
*Prlo*-DRB*58	1	ON
*Prlo*-DRB*59	1	NY
*Prlo*-DRB*62	15	NY, AL/GA, FL
*Prlo*-DRB*64	1	FL
*Prlo*-DRB*68	9	FL
*Prlo*-DRB*69	10	AL/GA, FL
*Prlo*-DRB*70	2	FL
*Prlo*-DRB*71	21	AL/GA, FL
*Prlo*-DRB*73	1	FL
*Prlo*-DRB*74	3	FL
*Prlo*-DRB*75	10	AL/GA, FL
*Prlo*-DRB*76	2	AL/GA, FL
*Prlo*-DRB*78	2	FL
*Prlo*-DRB*80	1	NY
*Prlo*-DRB*81	4	FL
*Prlo*-DRB*85	2	FL
*Prlo*-DRB*90	6	AL/GA, FL
*Prlo*-DRB*99	9	FL
*Prlo*-DRB*100	3	NY, AL/GA, FL
*Prlo*-DRB*102	4	AL/GA
*Prlo*-DRB*103	4	AL/GA

**Table 2:** Amino acid sequences of MHC DRB exon 2 alleles in raccoons.

Allele	* * * * * * ** ** * * * ** * * ** ** **
*Prlo*-DRB*01	NGTERVQLL VRNIYNGQED VRYDSDVGEH RAVTELGRPD AQYWNSQKDL MERRRAEVDT VCRHNYGVVE SFTVQR
*Prlo*-DRB*02	......RY. ..V...RE.Y ..F......F .........S .........F ..QK...... Y.......G. ......
*Prlo*-DRB*03	......RY. ..H....... .........Y .........E .......... ...T...... Y.......G. ......
*Prlo*-DRB*04	......RY. ..V....R.. ..F......F Q......... .........V V.QK..A... ........G. ......
*Prlo*-DRB*05	......R.. ..D....R.. ..F......F Q......... .E.......V V.Q....... ........G. ......
*Prlo*-DRB*06	......RY. ..V....R.. ..F......F .......... .........F ..QK..A... .......... ......
*Prlo*-DRB*07	......R.. ..D...RE.Y .......... ........QI .E.......F ..Q....... ........G. ......
*Prlo*-DRB*08	......RF. E.HF..R..F L.F......Y .......... .........F ..QN..A... Y.......G. ......
*Prlo*-DRB*09	......RY. ..H....... .........Y ........QI .E.L...... .......... ........G. ......
*Prlo*-DRB*10	......RY. ..V...RE.Y ..F......F .........S .........F ..QK..A... ........G. ......
*Prlo*-DRB*11	......RY. ..D....R.. ..F......Y .......... .E........ I.Q....... Y......... ......
*Prlo*-DRB*12	......R.. ..D......Y ..F......Y .........S ..N......F I.Q....... .......... ......
*Prlo*-DRB*13	......RF. ..Y......Y ..F....... .........S .........F ...T..A... Y.......G. ......
*Prlo*-DRB*14	......R.. T.D......Y ..F......F .......... .........V V.Q...A... Y.......G. ......
*Prlo*-DRB*15	......RY. ..E....R.. ..F......Y .......... .E........ I.Q...A... Y.......G. ......
*Prlo*-DRB*16	......RF. E.HF..R..F L.F......Y .......... .......... ..Q....... Y......... ......
*Prlo*-DRB*17	......R.. ..D....R.. ..F......Y .......... .E........ I.Q...A... Y.......G. ......
*Prlo*-DRB*18	......RF. ..V....R.. ..F......F Q......... .........V V.QK..A... Y.......G. ......
*Prlo*-DRB*19	......RY. ..V...RE.Y ..F......F .........S .........F ..QK..A... ........F. ......
*Prlo*-DRB*20	......R.. ..D...RE.Y .........Y .........S .......... I.Q....... ........G. ......
*Prlo*-DRB*21	......RF. ..Y....... ..F....... .......... .........F ...T..A... .......... ......
*Prlo*-DRB*22	......RF. ..V....R.. ..F......F Q......... .........V V.QK..A... ........G. ......
*Prlo*-DRB*24	......R.. ..H....... .........Y ........QI .E.L...... .......... ........G. ......
*Prlo*-DRB*25	......R.. .......... .......... .......... .......... .......... ........F. ......
*Prlo*-DRB*26	......RF. ..D....... ..F......F Q......... .........Y V.QK..A... Y.......G. ......
*Prlo*-DRB*27	......RV. T.Y......F ..F......F .......... .......... I.Q....... ........F. ......
*Prlo*-DRB*28	......R.. T.D......Y ..F......F .......... .........V V.Q....... Y......... ......
*Prlo*-DRB*29	......RY. ..D....R.. ..F......F Q......... .........F ..QK...... Y.......F. ......
*Prlo*-DRB*30	......RF. ..Y......Y ..F....... .......... .........F ..QN..A... Y.......G. ......
*Prlo*-DRB*31	......RF. E.HF..R..F L.F......Y .........T .........Y V.QK...... Y.......G. ......
*Prlo*-DRB*32	......R.. T.D......Y ..F......F .........S .........V V.Q...A... Y.......G. ......
*Prlo*-DRB*34	......R.. T.D......Y ..F......F .........S .........F .......... ........G. ......
*Prlo*-DRB*42	......RY. ..V....R.. ..F......F .......... .........F ..QN..A... Y......... ......
*Prlo*-DRB*43	......RF. ..M....R.. ..F......F .........T .........Y V.QK..A... Y.......G. ......
*Prlo*-DRB*47	......RY. ..D....R.. ..F......F .......... .........F ..QK..A... .......... ......
*Prlo*-DRB*48	......RY. ..V....R.. ..F......F .......... .........F ..QK..A... Y......... ......
*Prlo*-DRB*49	......RF. E.HF..R..F L.F......Y .......... .........F ..QN..A... Y......... ......
*Prlo*-DRB*50	......R.. T.D......Y ..F......F .......... .........F ..Q...A... Y.......G. ......
*Prlo*-DRB*51	......R.. T.D......Y ..F......F .......... .........V V.Q...A... Y......... ......
*Prlo*-DRB*52	......RY. ..V....R.. ..F......Y .......... .E........ I.Q....... Y......... ......
*Prlo*-DRB*53	......R.. T.D......Y ..F......F .........T .........F ..Q...A... Y......... ......
*Prlo*-DRB*54	......RY. ..H....... .........Y ........QI .E.L...... .......... .......... ......
*Prlo*-DRB*55	......RV. T.Y......F ..F......F .......... .......... I.Q....... .......... ......
*Prlo*-DRB*56	......R.. ..V...RE.Y ..F......F .......... .E.......V V.Q....... ........G. ......
*Prlo*-DRB*57	......RY. ..D....R.. ..F......F Q......... .........V V.QK..A... ........G. ......
*Prlo*-DRB*58	......R.. ......RE.Y .........Y .........S .......... I.Q....... ........G. ......
*Prlo*-DRB*59	......R.. .......R.. .........Y .........E .......... .......... .......... ......
*Prlo*-DRB*62	......RY. ..D....R.. ..F......F .......... .........F ..QK..A... ........F. ......
*Prlo*-DRB*64	......RY. .......R.. .........Y .......... .E........ I......... .......... ......
*Prlo*-DRB*68	......RN. ..D....R.. .........Y .......... .E........ I......... .......... ......
*Prlo*-DRB*69	......R.. .........F .......... ........QI .E.L.....F .......... ........G. ......
*Prlo*-DRB*70	......RF. ..D....... ..F......F .......... .........F ..QN..A... Y......... ......
*Prlo*-DRB*71	......RF. ..V....R.. ..F......F .......... .........F ..QN..A... Y.......G. ......
*Prlo*-DRB*73	......RY. ..D....R.. ..F......Y .......... .E........ I.Q....... Y.......G. ......
*Prlo*-DRB*74	......RY. ..D....R.. .........Y ........QI .E........ I......... ........F. ......
*Prlo*-DRB*75	......R.. ..D....R.. L.F......Y .......... .........F ..Q...A... Y.......G. ......
*Prlo*-DRB*76	......RN. ..D....R.. .........Y .......... .E........ I......... ........G. ......
*Prlo*-DRB*78	......RF. ..Y......Y ..F....... .........S .........F ...T..A... .......... ......
*Prlo*-DRB*80	......RF. ..V....R.. ..F......F Q.......QI TE.L...... ...T..A... ........F. ......
*Prlo*-DRB*81	......RY. ..V....R.. ..F......F Q......... .........F ..QN..A... Y.......G. ......
*Prlo*-DRB*85	......RF. ..Y......F ..F......Y .......... .........F ...T..A... Y......... ......
*Prlo*-DRB*90	......RN. ..V....R.. .........Y .......... .E........ I......... .......... ......
*Prlo*-DRB*99	......RF. ..V....R.. ..F...L..F .......... .........F ..QN..A... Y.......G. ......
*Prlo*-DRB*100	......R.. ..D....R.. .........Y .......... .E........ I......... ........G. ......
*Prlo*-DRB*102	......RY. ..D...RE.F .......... ........QI .E.......F ..Q....... Y.......G. ......
*Prlo*-DRB*103	......RY. ..D....R.. .........Y .......... .......R.. I......... ........F. ......

Dots indicate identity to the reference sequences. The putative peptide binding regions (PBR; Brown et al. [38]; Stern et al. [39]) are marked with asterisks.

**Figure 1:**
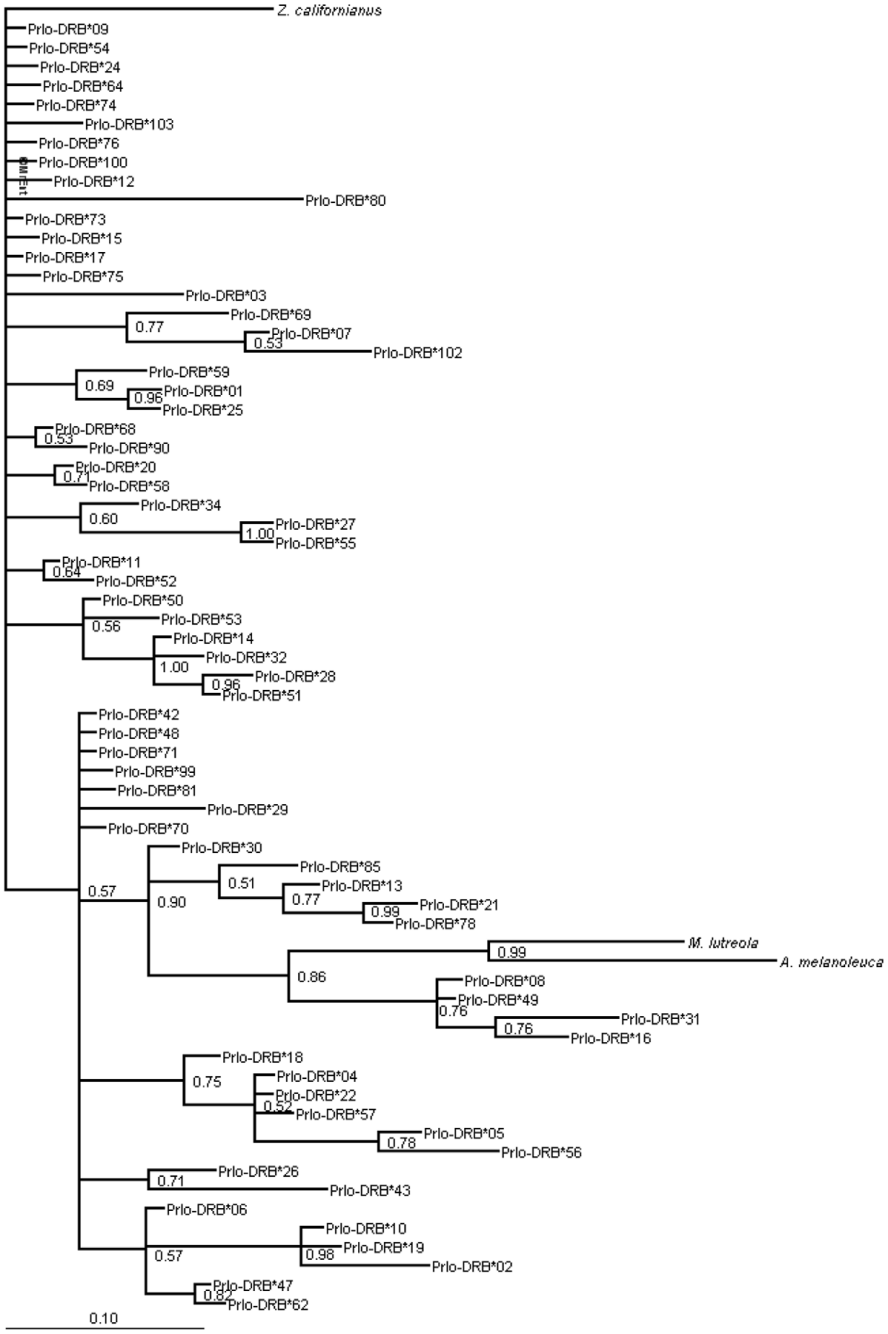
Bayesian phylogenetic relationship of raccoon MHC DRB exon 2. This tree was constructed using the best fit model from the JModel test [42], [43]. In addition to the 66 raccoon alleles, three MHC alleles belonging to other mammals were included as outgroups: *Zalophus californianus* (GenBank Accession AY491456), *Ailuropoda melanoleuca* (GenBank Accession EF125965), and *Mustela lutreola* (GenBank Accession EU263550).

Average pairwise K2P nucleotide distances and Poisson corrected amino acid distances were computed for all sites, PBR only and non-PBR only (Table 3). Phylogenetic relationship among raccoon MHC DRB exon 2 alleles were poorly resolved using both methods of phylogenetic analyses (Bayesian inference and K2P nucleotide distance); we therefore chose to only present the Bayesian tree as we concluded that the support for the branches were more accurate and reflective of the true phylogenetic relationships (Figure 1). When examining modes of selection acting on MHC DRB exon 2, there were signs of positive selection acting on this region of the genome with greater rates of nonsynonymous than synonymous substitutions found at the peptide binding regions (Table 4: P = 0.005, Z-test of positive selection).

**Table 3:** Average nucleotide and amino acid distances among raccoon MHC DRB exon 2 alleles.

K2P nucleotide distance			Poisson-corrected amino acid distance		
All sites	PBR	Non -PBR	All sites	PBR	Non-PBR
8.0 (1.2)	21.2 (4.2)	3.3 (0.9)	13.8 (3.0)	45.5 (12.4)	5.9 (2.4)

Standard errors (in parentheses) were obtained through 1000 bootstrap replicates. Distances were corrected for multiple substitutions using K2P model for nucleotide distances and Poisson distribution for amino acid differences. Putative peptide binding region (PBR) sites were those determined by Brown et al. [38] and Stern et al. [39] Distances are given as a percentage per site.

**Table 4:** Average rates of nonsynonymous substitutions per nonsynonymous site (d_N_) and synonymous substitutions per synonymous site (d_S_).

Sites	*d_N_*	*d*_S_	Z	P
All	7.1 (1.5)	7.6 (2.3)	−0.179	1.000
PBR	23.4 (5.9)	9.0 (2.3)	2.621	0.005
Non-PBR	3.1 (1.3)	3.7 (1.6)	−0.256	1.000

Results are given as percentages (stander errors obtained through 1000 bootstrap replicates in parentheses) and results of the Z-test for positive selection.

## Discussion

This study represents the first comprehensive investigation of MHC variation in the procyonidae family. Investigating MHC variation in raccoons will further our understanding of how the immune systems of this host species responds to invading organisms. Initial characterization was performed on the alpha region of the DR gene in raccoons. We determined that DRA exon 3 in raccoons is functionally monomorphic based on identical amino acid sequences of the three alleles. The finding that DRA exon 3 is monomorphic is similar to what has been observed in other mammalian species [45]. The lack of variation at DRA makes this locus inappropriate for studies of pathogen influence; therefore no further analyses were performed. However it is important to note that other exons in this locus may be polymorphic and may be used for studies of pathogen influence. Additionally, this locus can be utilized in future comparative studies.

We found that MHC DRB exon 2 is duplicated in raccoons, with between two and four alleles present in each individual. Duplication of MHC class II loci is common in mammalian groups, (e.g., sea lions [16], domestic cats [34], and chacma baboons [15]), with the majority of duplicated loci being functional [46]. Duplication of MHC loci also plays an important role in the adaptive evolution of organisms by increasing the number of alleles present in individuals, thereby allowing for the detection of a greater number of invading organisms [47]. We were unable to assign alleles to an individual locus as interlocus exchange is known to occur at MHC loci [48]. Therefore we considered all alleles to be representatives of the DRB locus for the phylogenetic analysis.

Our phylogenetic relationships (Figure 1) were poorly resolved using both Bayesian Inference and K2P nucleotide distance measures. This was expected given the relatively short sequence length and vast amount of polymorphism. Alleles clustered into two main clades, with the majority of alleles belonging to a single unresolved clade. The presence of multiple clades may relate to the different strains of the raccoon rabies virus present in raccoon populations in North America [49] given the strong selective force of rabies in these regions, or may be related to other selective pressures. Four *Prlo*-DRB alleles moderately cluster (86%) with the mink and giant panda outgroups suggesting possible trans-specific inheritance of some DRB sequences before divergence from a common ancestral sequence [15]. All alleles were also found to identify in the 80 percentile with DRB alleles from other animals, further suggesting that we were amplifying the DRB locus [44].

We found extremely high variation at MHC DRB exon 2 in raccoons with a total of 66 alleles discovered in 246 individuals analyzed (Table 2). The second exon of DRB is known to be highly polymorphic and the polymorphism is present at multiple base sites [50]. This is consistent with our finding of up to 36 nucleotide differences between alleles (*Prlo*-DRB*31 vs. *Prlo*-DRB*80). Doherty & Zinkernagel [51] proposed that polymorphism at MHC was related to the function of the peptide binding regions and ability to confer resistance to a wide range of pathogens. This implies that MHC polymorphism must be maintained by pathogen driven selection, [see 9] such as overdominance (heterozygote advantage) [6], [51] or frequency dependent selection (rare allele advantage/Red queen hypothesis) [52]. Either of these forms of pathogen driven selection may be driving polymorphism of MHC in raccoons.

We found evidence of positive selection acting on MHC with rates of nonsynonymous substitutions being 2.6 times greater than synonymous substitutions at the functionally important peptide binding region (PBR) (Table 4). The difference between rates of synonymous and nonsynonymous substitutions was much lower than what has been previously reported in other mammalian species (e.g., d_N_ was 5 times greater than d_S_ in spotted suslik [53]; d_N_ was 8.31 times higher than d_S_ in chacma baboons [15]). The lower difference between nonsynonymous and synonymous substitutions found in this study may be due to the addition of the peptide binding regions (PBRs) described by Stern et al. [39] which were added to include all the probable PBRs. In contrast, there was no significant difference between nonsynonymous and synonymous substitutions at the non-peptide binding regions. Beyond testing for positive selection, we also tested for significant departure from neutral expectations (d_N_ = d_S_) which has been proposed to be important for inferring the effects of selection acting on MHC diversity [9]. Significant deviation from neutrality was found (Z = 2.098, P =  0.038) at the PBR further supporting the idea that positive selection has been the strongest form of selection acting on MHC in raccoons.

Understanding variation of the immune response in raccoon is necessary as there is an ongoing epizootic of the raccoon variant of rabies in North America. Due to increased density of raccoons in urban areas, there is a higher risk of rabies transmission to humans, domestic animals and livestock [54], [55]. Although rabies was thought to be 100% lethal, thereby having no evolutionary potential, it has been illustrated that immunity may exist in raccoon populations [56]. The data presented here will form the basis of continued analyses into the spatial and temporal relationship of the raccoon rabies virus and the immunogenetic response in its primary host.
